# Endoscopic Findings and Their Association With Gender, Age and Duration of Symptoms in Patients With Dysphagia

**DOI:** 10.7759/cureus.11264

**Published:** 2020-10-30

**Authors:** Hafsa Rashid, Khush Bakht, Amna Arslan, Amna Ahmad

**Affiliations:** 1 Gastroenterology and Hepatology, Pakistan Emirates Military Hospital, Rawalpindi, PAK; 2 Internal Medicine, Shalimar Medical College, Lahore, PAK; 3 Internal Medicine, Fauji Foundation Hospital, Lahore, PAK; 4 Diagnostic Radiology, Shaikh Zayed Hospital, Lahore, PAK

**Keywords:** dysphagia, endoscopic findings, duration of symptoms

## Abstract

Introduction

Dysphagia is highly prevalent in patients with a history of recurrent acid peptic disease. Endoscopy is the mainstay of diagnostic workup of these patients to reach underlying cause and appropriate subsequent treatment. The objective of the study was to determine the frequency of various types of endoscopic findings in patients with dysphagia and the association of these findings with gender, age and duration of symptoms.

Methods

This cross-sectional study was carried out in the Department of Gastroenterology of a tertiary care hospital in Rawalpindi, Pakistan. A total of 137 patients who presented with a history of dysphagia for at least two weeks were enrolled in the study. Duration of symptoms was noted, and all patients underwent upper gastrointestinal endoscopy to find out the cause of dysphagia. Tissue biopsies were obtained, and further histopathological examination was performed to correlate the findings with symptoms of dysphagia.

Results

A total of 137 patients were enrolled for six months. The mean age of the patients was 56.9 ± 17.44 years, and the mean duration of symptoms was 15.96 ± 12.31 weeks. There were 65 (47.4%) males and 72 (52.6%) females in the study. Majority of them, 123 (89.8%), presented with a short duration of symptoms that varied between 2-24 weeks and were mainly middle-aged (31-60 years) and old-aged (61-80 years). The most commonly observed endoscopic findings were esophageal stricture in 25 (18.2%), achalasia cardia in 20 (14.6%), esophageal mass in 12 (8.8%) and reflux esophagitis in 7 (5%) patients. No association was seen between age, gender and duration of symptoms and findings on the endoscopy.

Conclusion

Dysphagia is associated with many endoscopic findings that are not related to demographic variables and must be evaluated earlier to reduce further morbidity and mortality.

## Introduction

Dysphagia is the symptom that describes the difficulty in swallowing due to any cause [1]. Numerous established reasons for dysphagia have been reported, but the prevalence varies in different regions. Various studies have reported dysphagia to range between 6-9% in other age groups. In patients above 50 years of age, dysphagia affects 16-22% of the population [2].

The most frequently performed procedure for initial diagnosis and evaluation of dysphagia of esophageal origin is upper gastrointestinal (GI) endoscopy [3]. It helps in the visualization of the entire esophagus directly and also aids in taking tissue for biopsy. It is essential to diagnose the cause of dysphagia early because it is linked with significant morbidity and mortality [4]. Upper GI endoscopy is a safe procedure and has a risk of complications of 1 per 1000 procedures approximately [3]. The most common complications seen are bleeding, infection, perforation and cardio-pulmonary issues [1,5].

The findings on endoscopy in patients with dysphagia are well known. In one literature review, it was found that in patients who had dysphagia, upper GI endoscopy revealed that 32.5% patients had normal findings, 25% had Schatzki's ring, 22.5% had malignant esophageal stricture, 10% had reflux esophagitis, and 5% had a benign stricture of esophagus [5-7]. In another study, it was found that 21% had a benign stricture of esophagus, and 14% of patients were diagnosed with achalasia [2].

Numerous studies have been carried out on the findings of endoscopy with dysphagia; however, the prevalence of these findings have not been well documented in local databases of Pakistan. Therefore, the current study aimed to determine the frequency of endoscopic findings in patients with dysphagia and the association of these findings with age, gender and duration of symptoms.

## Materials and methods

It was a cross-sectional study conducted at the Department of Gastroenterology and Hepatology, Pak Emirates Military Hospital, Rawalpindi, Pakistan for a period of six months from July 2018 to December 2018. A sample size of 137 was calculated using the Epi-Pen calculator based on the prevalence of dysphagia to be 9% in general population-based on previous studies (8). The patients were recruited using consecutive non-probability sampling. All patients presenting with dysphagia from age 18-80 years were included in the study. Patients with a previous history of upper gastrointestinal (GI) endoscopy and any past GI surgery were excluded from the study. The study was conducted following principles of ethical medical practice as laid down in the Declaration of Helsinki after obtaining its formal approval from the Ethical Committee of the same institution.

Patients fulfilling the inclusion criteria were recruited in the study after obtaining informed written consent. The patients were assessed with adequate history, thorough examination and investigations and findings were noted down on a pre-designed proforma. Endoscopy was performed by the same consultant gastroenterologist (HR), and biopsies were taken where required and were sent for histopathological analysis. The results of the analysis were recorded on the proforma and were subjected to statistical analysis. Radiography was advised in all cases to see radiological findings in patients with dysphagia.

The data were entered and analyzed through Statistical Package for Social Sciences (SPSS version 24.0, IBM Statistics, IL, USA). Quantitative data such as age and duration of symptoms were presented as mean and standard deviation. Qualitative data such as gender, age groups, duration of symptoms, physical findings, radiological findings, endoscopic findings and biopsy findings were calculated as frequencies and percentages. Chi-square test was used to compare qualitative data, whereas a student's t-test was used for quantitative variables. A p-value of ≤ 0.05 was considered as statistically significant.

## Results

A total of 137 patients were enrolled in this study. The mean age of the patients was 56.9 ± 17.4 years, and the mean duration of symptoms was 16.0 ± 12.3 weeks. There were 65 (47.4%) males and 72 (52.6%) females in the study. Distribution of patients in various age groups and duration of dysphagia is shown in Table 1. Findings of the patients on physical examination and their radiographic investigations are presented below (Table 2, 3).

**Table 1:** Demographic variables of the study population

Study variables	Number of Patients [ n (%)]	p-Value (chi-square test)
Gender		
Male	65 (47.4%)	0.45
Female	72 (52.6%)	
Age groups (years)		
Young age (18-30)	18 (13.1%)	
Middle age (31-60)	57 (41.6%)	0.073
Old age (61-80)	62 (45.3%)	
Duration of dysphagia (weeks)		
Short duration (2-24)	123 (89.8%)	0.988
Long duration (25-48)	13 (9.5%)	
Extremely long duration (> 48)	1 (0.7%)	

**Table 2:** Physical examination findings of patients with dysphagia ILD- Interstitial lung disease

Physical findings	n (%)
Emaciated	10 (7.3%)
Hyperthyroid	1 (0.7%)
Hypothyroid	2 (1.5%)
Known case of celiac	1 (0.7%)
Known case of ILD	1 (0.7%)
Loss of weight	39 (28.5%)
Pallor	72 (52.6%)
Paralysis	7 (5.1%)
Parkinsonism	2 (1.5%)
Ptosis	1 (0.7%)
No findings	1 (0.7%)

**Table 3:** Radiological findings in patients with dysphagia

Radiological findings	n (%)
Achalasia cardia	19 (13.9%)
Lower esophageal narrowing	1 (0.7%)
Dilated esophagus	5 (3.6%)
Hiatus hernia	1 (0.7%)
Stricture	30 (21.9%)
Diverticulum	4 (2.9%)
Ulcer	2 (1.5%)
Stricture (malignant)	1 (0.7%)
Nutcracker esophagus	1 (0.7%)
No finding	73 (53%)

Various endoscopic findings were seen in 57 (41.6%) patients in the elderly (61-80 years) as described below. The most common findings in this age group were achalasia cardia in 10 (7.3%), esophagitis in 8 (5.8%) and esophageal masses in 7 (5.1%). In middle age group (31-60 years), endoscopic findings were present in 50 (36.5%) patients and the most common findings were esophageal stricture 14 (10.2%), esophageal mass 5 (3.6%) and achalasia in 5 (3.6%). Among young age group (18-30 years), positive endoscopic findings were observed in 15 (11.0%) patients and the most common findings were of achalasia cardia in 5 (3.6%), corrosive stricture in 4 (2.9%) and esophageal stricture in 3 (2.2%). Despite variability in presentations at different age groups, there was no significant association found between age and endoscopic findings (Table 4).

**Table 4:** Findings of upper gastrointestinal endoscopy in patients with dysphagia GEJ- Gastroesophageal Junction

Endoscopic Findings	n (%)
Normal findings	15 (10.9%)
Esophageal web	4 (2.9%)
Peptic stricture	4 (2.9%)
Lax LES	1 (0.7%)
Gastric mass	2 (1.5%)
Reflux esophagitis	7 (5.1%)
Esophageal growth	1 (0.7%)
GEJ growth	5 (3.6%)
Zenkar diverticulum	1 (0.7%)
Multiple ulcers in esophagus	1 (0.7%)
Growth hypopharynx	3 (2.2%)
Esophageal mass	12 (8.8%)
Esophageal candidiasis	4 (2.9%)
Superficial ulcers in esophagus	1 (0.7%)
Superficial ulcers in larynx and pharynx	1 (0.7%)
Peptic stricture at 35cm	1 (0.7%)
Diffuse hypertrophy of stomach	1 (0.7%)
Esophagitis	1 (0.7%)
Globus hystericus	1 (0.7%)
Cockscrew esophagus	1 (0.7%)
Hiatus hernia and barrets esophagus	2 (1.5%)
Foreign body	1 (0.7%)
Achalasia cardia	20 (14.6%)
Growth of larynx	2 (1.5%)
Schatzki ring	2 (1.5%)
Nodular mucosa of stomach	1 (0.7%)
Growth of pharynx, larynx and esophagus	1 (0.7%)
Nutcracker esophagus	1 (0.7%)
Barrets esophagus Stricture at GEJ	3 (2.2%) 1 (0.7%)
Corrosive stricture	4 (2.9%)
Pharyngeal mass	1 (0.7%)
Esophageal mucosal bulge	1 (0.7%)
Hiatus hernia with cameron lesion	1 (0.7%)
Esophageal stricture	25 (18.2%)
Esophageal diverticulum	4 (2.9%)

With regards to gender, 60 males and 62 females had positive findings on upper GI endoscopy (p-value > 0.05). The common results in males were esophageal stricture 14 (10.2%), achalasia cardia in 10 (7.3%), esophageal mass in 5 (3.6%) and gastro-oesophagal junctional (GEJ) growth in 4 (2.9%). In females, the typical findings were esophageal stricture 11 (8%), achalasia cardia 10 (7.3%), esophageal mass 7 (5.1%) and corrosive stricture in 4 (2.9%). There was no statistically significant association between gender and the findings on endoscopy in patients who presented with dysphagia.

In patients who presented with short duration of dysphagia, i.e. from 2 to 24 weeks, the most common findings were esophageal stricture in 22 (16.1%) patients, achalasia cardia in 19 (13.9%), esophageal mass in 9 (6.6%), reflux esophagitis in 7 (5.1%), GEJ growth in 5 (3.6%) and esophageal stricture, esophageal candidiasis, esophageal diverticulum and peptic stricture in 4 (2.9%) each. Among patients who presented with long duration of dysphagia, i.e. from 25-48 weeks, common endoscopic findings were of esophageal stricture in 3 (2.2%) and corrosive stricture in 2 (1.5%) patients. No endoscopic finding was found in patients who presented with too long duration dysphagia, i.e. > 48 weeks. No significant association was found between the duration of dysphagia and endoscopic findings as indicated by the p-value of >0.05. The results of tissue histopathological examination are presented below (Table 5).

**Table 5:** Findings of histopathological examination in dysphagia patients

Biopsy findings	n (%)
Squamous cell carcinoma	14 (10.2%)
Adenocarcinoma	1 (0.7%)
Intestinal metaplasia	1 (0.7%)
Benign stricture	2 (1.5%)
Achalasia cardia	6 (4.4%)
Zenkar diverticulum	1 (0.7%)
Peptic stricture	4 (2.9%)
Drug induced esophagitis	1 (0.7%)
Esophageal candidiasis	1 (0.7%)
Reflux esophagitis	1 (0.7%)
Biopsy not done	105 (76.6%)

## Discussion

Dysphagia is a frequent complaint of patients presenting in GI clinics [7-10]. The causes of dysphagia of esophageal origin that are identified commonly are benign and malignant strictures of esophagus, reflux esophagitis, Schatzki's ring, compression occurring externally from a malignancy, disorders of motility, scleroderma and achalasia [11-16]. In some population-based studies, the rates of prevalence of dysphagia have been reported to be 17-25% with a peak in symptoms at the age of 40-49 years for both gender [17,18]. More commonly, it is seen in old age patients and in those who were hospitalized [19]. The modality of choice for making a diagnosis of dysphagia is upper gastrointestinal endoscopy and has a good safety profile too [20,21].

The most common findings in the study on endoscopy in patients who presented with dysphagia were esophageal stricture in 25 (18.2%), achalasia cardia in 20 (14.6%), esophageal mass in 12 (8.8%), reflux esophagitis in 7 (5.1%), esophageal diverticulum in 4 (2.9%), corrosive stricture in 4 (2.9%), esophageal candidiasis in 4 (2.9%), esophageal web in 4 (2.9%) and peptic stricture in 4 (2.9%).

Esophageal stricture was seen more in patients who were of middle age (14,10.2%) followed by in old age patients above 60 years of age (8, 5.8%). It was more frequent in males (14, 10.2%) compared to females (11, 8.0%) and was more common in patients who presented with short duration of dysphagia (2-24 weeks) as compared to patients who presented with long term (> 48 weeks) (22, 16.1% versus 3, 2.2%). This implies that esophageal stricture is mainly found in middle-aged males with short duration of symptoms of dysphagia. In a study by Eslick et al. [5] it was found to be the second most common cause of dysphagia (22, 17.3% patients). The study revealed that it was more common in 30-50 years old patient with long duration of symptoms (>24 weeks). The findings of the study were similar to our study in terms of age of the patients. Both these studies revealed that middle-aged people had more esophageal strictures even in the presence of symptoms of dysphagia for a relatively shorter duration of time.

The second most common finding on endoscopy was of achalasia cardia. It was commonly seen in patients who had a short duration of symptoms (<24 weeks), i.e. in 19 (13.9%). Only one (0.7%) patient with a duration of symptoms of >24 weeks had achalasia cardia. Both males and females had similar findings of achalasia cardia, i.e. 10 (7.3%) each. It was more prevalent in the old age group of >60 years age in a frequency of 10 (7.6%), whereas young and middle-aged patients had similar rates of achalasia cardia, i.e. in 5 (3.6%) each. In one study, achalasia cardia was found to be the 4th most common cause of dysphagia and was seen in 9.4% patients, and the majority of the patients were of age <30 years, i.e. 43.8%, 11.1% were of age 30-50 years and no patient above 50 years had achalasia cardia [5]. It was seen to affect males more and was frequently encountered in patients with short duration of symptoms. In another study, similar results were found in terms of gender and time of symptoms, but it was found to occur more frequently in old age patients who were >50 years [6]. The current study revealed similar findings in terms of duration of symptoms, and the higher prevalence of achalasia cardia in old age was similar to the results of the study reported by Khan et al. [6].

Thirdly, esophageal mass was found as a common finding. It was more common in old age people (61-80 years), i.e. in seven (5.1%) patients followed by in middle-aged patients 5 (3.6%) patients. It was more commonly seen in females seven (5.1%) and was more frequent in patients with dysphagia of short duration, i.e. 9 (6.6%). Yahya et al. reported that in patients with dysphagia, esophageal mass was a common cause, and it was reported to occur in 28.4% patients [7]. In our study, esophageal mass was found in 8.8% of patients. Findings similar to our study in terms of esophageal mass were reported by Gouda et al. as well [3].

A fourth common finding was that of reflux esophagitis in 7 (5.1%) patients. Reflux esophagitis was slightly more frequent in females than males, i.e. four (2.9%) and three (2.2%) respectively. It was more frequent in patients with a short duration of symptoms, i.e. in 7.1% and was more frequent in middle-aged people, i.e. in 4 (2.9%). Findings from other studies revealed similar prevalence rates in terms of gender. However, other studies showed that reflux esophagitis was more common in old age patients with duration of dysphagia for >24 weeks [3-5,17].

This study had certain limitations. First of all, the study was carried out in a single-centre, so the results cannot be generalized to the general population with different dietary habits. Secondly, the sample size was small, so it cannot be taken as a representation of the whole population. Thirdly, endoscopy was only used for diagnosis, and the therapeutic use of the endoscopy was not evaluated. Lastly, the database did not provide thorough details for evaluation of causes of these findings and additional testing for further diagnostic confirmation was not done. Future studies on large sample size with subjects from various backgrounds and different dietary habits must be carried out in order to look for the association between demographics and findings on endoscopy as well as with other factors that can play an essential role in the pathogenesis of dysphagia.

## Conclusions

Based on results, the current study concluded that the most common findings on endoscopy in patients with dysphagia were esophageal stricture, achalasia cardia, esophageal mass and reflux esophagitis. No significant association was found between the endoscopic findings and age, gender and duration of dysphagia. Upper gastrointestinal endoscopy must be carried out in all patients who present with dysphagia in order to look for causes like esophageal stricture, achalasia cardia, esophageal mass and reflux esophagitis , which if diagnosed earlier and treated promptly can reduce the risk of further morbidity and mortality associated with dysphagia. Endoscopy of the upper gastrointestinal tract is a safe, convenient and effective procedure for establishing the diagnosis and providing information about different treatment strategy.
